# Purity Assessment of Dinotefuran Using Mass Balance and Quantitative Nuclear Magnetic Resonance

**DOI:** 10.3390/molecules28093884

**Published:** 2023-05-04

**Authors:** Xianjiang Li, Wei Zhang, Xiao Li, Shukun Zhou, Mengling Tu, Yunxiao Zhu, Hongmei Li

**Affiliations:** 1 Key Laboratory of Chemical Metrology and Applications on Nutrition and Health for State Market Regulation, Division of Metrology in Chemistry, National Institute of Metrology, Beijing 100029, China; 2 State Key Laboratory of Heavy Oil Processing, College of Chemical Engineering and Environment, China University of Petroleum, Beijing 102249, China

**Keywords:** dinotefuran, certified reference material, mass balance, quantitative nuclear magnetic resonance spectroscopy

## Abstract

Dinotefuran (DNT) belongs to the third-generation neonicotinoid pesticides, which are among the most common residuals in a variety of food commodities. To guarantee accurate quantification and traceability of results in food samples, certified reference materials (CRMs) are the indispensable benchmark. In this work, a DNT CRM was characterized and its purity was assessed by two independent methods, including mass balance (MB) and quantitative nuclear magnetic resonance spectroscopy (qNMR). The mass fraction of moisture was 0.33 mg/g, the inorganic impurity was 0.01 mg/g, and no detectable organic solvent was detected. Benzoic acid was chosen as the internal standard for qNMR. Its mass fraction was 997.9 mg/g and 992.9 mg/g by MB and qNMR, respectively. Eventually, the DNT CRM was assigned a mass fraction of 995 mg/g, with expanded uncertainty of 5 mg/g (*k* = 2). This CRM can be used to prepare calibrant solutions and is applicable to national routine monitoring of DNT residuals in agro-products and food.

## 1. Introduction

Neonicotinoid (NEO) insecticides are very efficient in pest control and have accounted for one-fourth of the global insecticide market since 2019 [1]. This family includes imidacloprid, acetamiprid, nitenpyram, thiamethoxam, clothianidin, thiacloprid, dinotefuran (DNT), cycloxaprid, and some new members. Dinotefuran is the common name for (RS)-1-methyl-2-nitro-3-(tetrahydro-3-furylmethyl) guanidine, with the CAS registry number 165252-70-0. As the first commercially available member of the third-generation neonicotinoids (furanicotinyl class) by Mitsui Chemicals [2], DNT has no chloride and sulfur elements and has the same good broad-spectrum insecticidal activity [3]. It is postulated that DNT affects the insect’s nicotinic acetylcholine binding in a mode different from that of the other NEOs. Additionally, it has little neonicotinoid effect on fruit quality and a lower ecological risk [4]. The formulated DNT products are widely applied to soil, foliage, nursery boxes, and paddy water by drench, spray, and broadcast treatments. After two decades of global use, DNT has been one of the most frequently detected residual insecticides in food commodities, including melons [5], oranges [6], cabbage [7], honey [8], cantaloupes [9], water [10], etc. Because it has good water solubility, it can penetrate into food tissues. With its heavy usage and wide contamination, deep safety concerns arise with DNT. Further studies have shown that DNT and metabolites already exist in human teeth [11], urine [12], and sera [13]. The Joint FAO/WHO Meeting on Pesticide Residues report estimated that the acceptable daily intake for humans was 0–0.2 mg/kg bw. France has banned the usage of five NEOs since 2018 [14], and DNT is promising to take their roles. The National Institutes of Health reported that NEOs could bind to the nicotinic acetylcholine receptor in mammals and lead to serious neurotoxicity [15]. A recent report showed that exposure to DNT and metabolites may also increase the possibility of liver cancer [16]. China issued a national food safety standard (GB 2763-2021) to forbid its intensive use, and DNT residues were strictly restricted in 52 kinds of foodstuff. The routine monitoring of residual DNT is indispensable for food safety and human wellbeing.

Because of the polarity of DNT (logP = −0.55) [17], liquid chromatography–ultraviolet detection (LC-UV) [18] and liquid chromatography–mass spectrometry (LC-MS) [19,20,21] are widely used techniques. In addition to the detection method, calibrants are also indispensable for accurate results. In China, thousands of vegetable and fruit samples were collected from markets and tested in authorized laboratories for DNT-related health risk assessment. The National Metrology Institute shoulders the responsibility of developing CRMs to guarantee accuracy and traceability [22]. Therefore, a National Key R&D Program of China (2019YFC1604801) was launched to develop pesticide CRMs. DNT was included because of its importance for food safety concerns.

Purity CRM are located at the top of the metrological traceability chain and are highly necessary to guarantee the reliability of detection. As a result, purity assessment of a DNT CRM is a prerequisite for calibrant solutions and following reliable measurements. The mass balance (MB) and quantitative nuclear magnetic resonance (qNMR) methods are internationally recognized primary methods for the purity assessment of organic materials. MB is an indirect method whereby the mass fraction can be directly traced back to the International System of Units (SI) [23]. By this strategy, the mass fraction can be calculated by subtracting all of the impurities from 1000 mg/g, typically including related structural impurities, water, residual organic solvents, and inorganic impurities. The accuracy of MB strongly depends on baseline separation and the method sensitivity of each impurity. Meanwhile, qNMR is a direct method whereby the mass fraction is assigned with a well-characterized SI-traceable internal standard [24]. Hydrogen is the most widely used element for qNMR. The selection of the internal standard and quantitative hydrogen is critical for this method. Value assignments of organic CRMs commonly adopt a combined methodology of MB and qNMR to provide a solid result.

In this work, an extensive purity assessment of a DNT CRM was implemented with both MB and qNMR methods for the first time. In the MB method, structurally related impurities were determined by LC-UV. The other impurities (i.e., volatile organic compounds, water, and inorganic elements) were quantified by purge-trapping gas chromatography–mass spectrometry (PT-GC-MS), Karl Fischer titration, thermogravimetric analysis (TGA), and inductively coupled plasma mass spectrometry (ICP-MS). In the qNMR method, the mass fraction was calculated by the selected hydrogen peaks from benzoic acid (internal standard) and the sample. This DNT CRM could trace to the SI unit, and the associated uncertainty contribution of the two methods was carefully evaluated. As far as we know, this is the first reported pure CRM of DNT.

## 2. Results and Discussion

### 2.1. Qualitative Characterization

**Figure 1:**
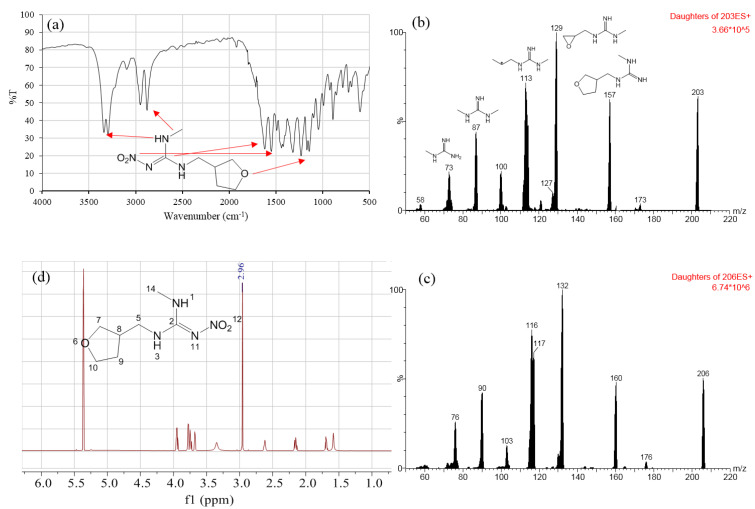
Characterization of DNT: (**a**) IR spectrum, (**b**) MS/MS spectrum of DNT, (**c**) MS/MS spectrum of DNT-d_3_, (**d**) ^1^H NMR spectrum.

The aim of qualitative characterization was to verify the identity of the DNT material. The IR spectrum shown in Figure 1a is consistent with the reported spectrum from Jiang’s book [25]. The characteristic absorption peaks can be attributed to typical functional groups in DNT. In detail, the peak at 3300 cm^−1^ was attributed to -NH- stretching vibration, the peak at 2900 cm^−1^ to -N-CH_3_ stretching vibration, the peak at 1610 cm^−1^ to -C=N- stretching vibration, the peak at 1550 cm^−1^ to -NO_2_ stretching vibration, and the peak at 1143 cm^−1^ to -C-O-C stretching vibration.

For the full-scan spectrum, the precursor ions (*m/z* 203 and 206) of DNT and DNT-d_3_ were detected by Waters TQ-S. Then, the precursor ions were fragmented to record the product ions (Figure 1b). The typical product ions of DNT were 157 for [M+H-NO_2_]^+^ and 129 for [M+H-C_2_H_4_-NO_2_]^+^, in accordance with spectrum from MassBank with the ID EQ310801 [26]. Additionally, the typical daughter ions of DNT-d_3_ were 160 and 132 (Figure 1c). DNT-d_3_ had three deuterium atoms in the methyl group. All of the captured ions in DNT-d_3_ were 3 *m/z* heavier than those of DNT. This indicated that the deuterium guanidine structure was quite stable. The mass deviations of the daughter ions helped in the elucidation of the fragmentation process. The chemical structures of typical ions are demonstrated in Figure 1b.

Figure 1d shows the ^1^H spectrum of DNT. The proton shifts were H-5 (3.32 ppm, 2H), H-7 (3.61 ppm, 1H; 3.74 ppm, 1H), H-8 (2.58 ppm, 3H), H-9 (1.65 ppm, 1H; 2.10 ppm, 1H), H-10 (3.70 ppm, 1H; 3.89 ppm, 1H), and H-14 (2.96 ppm, 3H). Additionally, the chemical shift of the residual solvent in methylene chloride-d_2_ was 5.32 and 1.68 ppm. The ^13^C spectrum of DNT is shown in Figure S2. The chemical shifts were 158.68, 71.00, 67.58, 44.84, 38.34, 29.58, and 28.01 ppm, which matched the predicted spectrum from MestReNova (V12.0).

The above results are in accordance with the findings of a previous report [2], so the candidate material was DNT.

### 2.2. Quantitative Analysis by Mass Balance

#### 2.2.1. Contents of Structurally Related Impurities

From the above acquired fragment ions and calculated neutral losses, some hints were useful for the structural elucidation. Sodium adducts and non-covalent dimer ions were observed for all peaks. The DNT and other peaks had many similarities in the fragment ions. The ion *m/z* 87.0796 appeared in all analytes.

The challenge of determining structurally related impurities was the baseline separation of the main component from every impurity [27]. Different columns and combinations of eluents with various gradient programs were intensively explored to baseline separate all impurities as much as possible. The details of the impurity identification and quantification were included in our previous work [28]. At last, two byproduct impurities and one stereoisomer were identified. Because these impurities had quite similar chemical structures to DNT, relative quantitation was carried out by the normalization of the peak area in the chromatogram. The maximum adsorption wavelengths were 267 nm, 268 nm, 270 nm, and 269 nm for four peaks. The purity of DNT was 99.82%, with a relative standard deviation (RSD) of 0.004%, under the wavelength of 269 nm. The calculated standard uncertainty was 0.004%, including the contribution from repeatability (*u*_1_) and the difference in the UV response factor (*u*_2_) (Table S1). The LOD and LOQ were 5 μg/L and 20 μg/L for the developed LC method, respectively. Good linearity was achieved for DNT, with concentrations ranging from 20 μg/L to 400,000 μg/L. Analogue elucidation not only provides the mass fraction in MB but also guides the selection of the quantitative hydrogen peak in qNMR.

#### 2.2.2. Mass Fraction of Residual Organic Solvent

The residual organic solvent was determined by the PT-GC-MS method. Pure water was tested as a blank sample. After background subtraction, the results showed that no detectable organic solvent was present in the DNT material.

#### 2.2.3. Mass Fraction of Water

From the synthesis pathway of DNT, water is used as a solvent for some polar precursors [2]. DNT has good solubility in methanol; therefore, direct addition instead of oven-assisted Karl Fischer titration was used. The blank value was determined with dummy addition to compensate for the adventitious water introduced during the sample loading, which was then subtracted from the mass of water determined in the DNT sample. From the result, the mass fraction of water was 0.33 mg/g, with an RSD of 6.0%. This value was proposed in rectangular distribution, and the standard uncertainty was 0.190 mg/g. The trace water content may come from residual solvent or moisture in the air during storage.

#### 2.2.4. Determination of the Mass Fraction of Inorganic Impurities

As shown by the TGA curve in Figure S1, the weight of the DNT was kept constant after 650 °C. The final weight was almost zero, and the total content of inorganic impurities was below the instrument sensitivity (0.1 μg). This indicated the presence of no detectible inorganic impurities in DNT according to the TGA method. Meanwhile, for ICP-MS, it had better sensitivity. A semi-quantitative analysis mode was performed for preliminary screening for the inorganic elements. As shown in Table S2, the total mass fraction of inorganic impurities was only 0.01 mg/g according to the ICP-MS method. The associated standard uncertainty was estimated as 0.005 mg/g.

#### 2.2.5. Mass Fraction by MB

Using the MB method, the mass fraction of DNT was calculated by subtracting the contents of all impurities from 1000 mg/g, as shown in Equation (1):

(1) $$P_{MB}=\left[1-\left(P_{W}+P_{NV}+P_{OS}\right)\right]\times P_{HPLC}$$

where *P_HPLC_* is the content of DNT measured by LC-UV (99.82%), *P_W_* is the content of water (0.33 mg/g), *P_OS_* is the content of residual organic solvent (0 mg/g), and *P_NV_* is the content of inorganic impurities (0.01 mg/g). Ultimately, the mass fraction of DNT was determined to be 997.9 mg/g.

### 2.3. Quantitative Analysis by qNMR

The equation for qNMR was as follows:

(2) $$P_{qNMR}=\frac{I_{DNT}}{I_{Std}}\frac{n_{Std}}{n_{DNT}}\frac{M_{DNT}}{M_{std}}\frac{m_{Std}}{m_{DNT}}P_{std}$$

where *m_std_* and *m_DNT_* are the weight of benzoic acid and DNT, respectively, *M_std_* and *M_DNT_* are the molecular weight of benzoic acid and DNT, respectively, *n_std_* and *n_DNT_* are the number of hydrogens in the quantification peaks of benzoic acid and DNT, respectively, *I_std_* and *I_DNT_* are the integrated signal intensities of the quantification peaks of benzoic acid and DNT, respectively, and *P_std_* and *P_qNMR_* are the mass fraction of benzoic acid and DNT, respectively.

**Figure 2:**
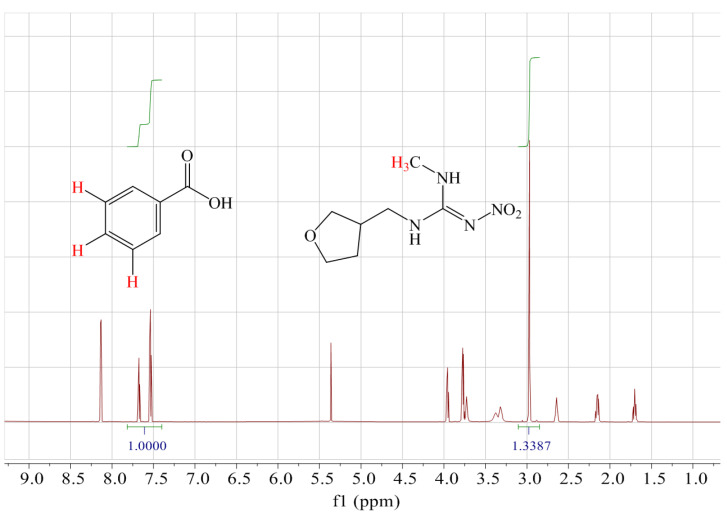
H-NMR spectra of DNT and benzoic acid.

The mass fraction of DNT was directly assigned to the benzoic acid CRM using the relative integral ratios of the selected hydrogen signals of benzoic acid and DNT. Because the second impurity had one more furan ring in the methyl group of DNT, this methyl hydrogen was a good choice for quantification without overlapping with the second impurity. Moreover, the aromatic hydrogen of benzoic acid (7.39–7.82 ppm 3H) and the methyl hydrogen of DNT (2.96 ppm 3H) were isolated from the other peaks and exhibited good peak symmetry for precise integration in Figure 2. Hence, these hydrogen peaks were chosen for quantification. The mass fraction of DNT was 992.9 mg/g, with an RSD of 0.03% in six duplicates.

### 2.4. Value Assignment

It is desirable to obtain consistent values from two independent methods, while the mass fraction determined by the MB method was a little higher than that determined by the qNMR method. This situation is common, because impurities are hardly comprehensively quantified in the MB method [29]. Finally, the mass fraction of the DNT CRM was 995 mg/g, taking the mean value of the MB and qNMR methods.

### 2.5. Uncertainty Evaluation

The uncertainty was evaluated according to the metrological technical specification for purity assessment of certified reference materials–organic purity certified reference materials (JJF 1855-2020) and the general and statistical principles for characterization of reference materials (JJF 1343-2022). In detail, the contribution to the uncertainty of characterization was from the methods of qNMR and MB, with their main influencing parameters. For the MB method, the combined standard uncertainty (*u_MB_*) was obtained by quadratic combination of the uncertainties from all detectable impurities, as shown in Equation (3):

(3) $$u_{MB}=P_{MB}\;\times \sqrt{u_{rel,HPLC}^{2}+\frac{u_{W}^{2}+u_{NV}^{2}+u_{OS}^{2}}{{\left(1-P_{W}-P_{NV}-P_{OS}\right)}^{2}}}$$

where u*_rel,HPLC_* is the relative uncertainty from LC-UV (0.004%), u*_W_* is the uncertainty from the water content (0.190 mg/g), u*_NV_* is the uncertainty from inorganic impurities (0.005 mg/g), and u*_OS_* is the uncertainty from residual organic solvent (0 mg/g). At last, the uncertainty of MB was 0.2 mg/g.

For qNMR, the uncertainty budget of DNT was 0.3 mg/g, originating from Equation (4):

(4) $$u_{qNMR}=P_{qNMR}\sqrt{{\left(\frac{u\left(I_{TMX}/I_{std}\right)}{I_{TMX}/I_{std}}\right)}^{2}+{\left(\frac{u\left(M_{TMX}\right)}{M_{TMX}}\right)}^{2}+{\left(\frac{u\left(M_{std}\right)}{M_{std}}\right)}^{2}+{\left(\frac{u\left(m_{TMX}\right)}{m_{TMX}}\right)}^{2}+{\left(\frac{u\left(m_{std}\right)}{m_{std}}\right)}^{2}+{\left(\frac{u\left(P_{std}\right)}{P_{std}}\right)}^{2}}$$

These two methods were regarded as having unequal precision. So, the standard uncertainty ($u_{char}$) of characterization was half of the square root of the sum of squares of the purity bias and uncertainty from the two methods, as shown in Equation (5):

(5) $$u_{char}=\sqrt{{\left(P_{MB}-P_{qNMR}\right)}^{2}+u_{MB}^{2}+u_{qNMR}^{2}}∕2$$

Finally, the standard uncertainty of DNT characterization (*u_char_*) was 3 mg/g. The details are described in Table 1. The expanded uncertainty *u_char_* was calculated by multiplying the *u_char_* by the cover factor (*k* = 2) at a confidence level of 95%. As a result, the expanded uncertainty of the DNT characterization was 5 mg/g (*k* = 2).

## 3. Materials and Methods

### 3.1. Chemicals and Materials

HPLC-grade acetonitrile (ACN) was obtained from Merck (Darmstadt, Germany). The DNT candidate material and DNT-d_3_ were bought from Toronto Research Chemicals (Nanjing, China) and Alta Scientific (Tianjin, China), respectively. The stock solution of DNT was prepared at a concentration of 0.4 mg/mL with ACN and stored at −20 °C in the dark. Water was supplied by Hangzhou Wahaha Group Co., Ltd. (Zhejiang, China). KBr for infrared (IR) was supplied by Aladdin (Shanghai, China). Methylene chloride-d_2_ was obtained from Sigma-Aldrich (St. Louis, MO, USA). The water content (GBW13517, 50.7 mg/g, *U* = 0.6 mg/g, *k* = 2) and benzoic acid (GBW06148, 99.991%, *U* = 0.024%, *k* = 2) CRMs were obtained from the National Institute of Metrology (Beijing, China). The tuning solution (part no. 5184-3566) and calibration standard (part no. 8500-6940) for ICP-MS were obtained from Agilent Technology (Tokyo, Japan).

### 3.2. Instruments

A Shimadzu LC-20A (Kyoto, Japan) was used for the analysis of the main components and structurally related impurities. It was equipped with an Agilent ZORBAX Eclipse plus C_8_ column (5 μm particle size, 4.6 mm × 250 mm). A Waters UPLC TQ-S system (Manchester, UK) was used for qualitative analysis. The residual organic solvent was quantified on a Thermo Scientific Trace 1310-TSQ 8000 EVO (Austin, TX, USA) with a Lumin AQUATek 100 purge and trap concentrator (Mason, OH, USA). Separation was performed on a DB-624 column (30 m × 0.320 mm × 1.80 μm). The moisture content was determined using a Mettler-Toledo C30S Karl Fischer titrator (Greifensee, Switzerland). TGA tests were carried out on a PerkinElmer Pyris 1 (Waltham, MA, USA), and ICP-MS was conducted on an Agilent 8800 (Tokyo, Japan). IR was tested using a PerkinElmer 100 N (Waltham, MA, USA). A Bruker Ascend^TM^ 800 NMR nuclear magnetic resonance spectrometer (Billerica, MA, USA) was applied for characterization and quantification. A quadruple inverse 5 mm CPQCI cryo-probe was equipped with for the ^1^H spectrum. The Bruker NMR software TopSpin 3.1 was used for data processing. Samples were weighed using a Mettler-Toledo XP205 or UMX2 balance (Greifensee, Switzerland). An IKA HS260 was used for material homogenization (Staufen, Germany).

### 3.3. Qualitative Characterization

#### 3.3.1. IR Spectrometry

KBr crystal was dried in advance and milled with DNT to form a homogeneous mixture. Then, the powder mixture was pressed into a tablet for IR spectroscopy. Data were collected in the wavenumber range of 600–4000 cm^−1^, with 24 accumulations, and then compared with the reported spectra from the literature.

#### 3.3.2. LC-MS/MS Analysis

DNT and DNT-d_3_ were analyzed with the UPLC TQ-S system. The solution was directly injected via the Intellistart^TM^ system, with a constant flow (10 μL/min). The instrument parameters were operated as described in a previous report [7]. First, a full scan was carried out to find their precursor ions in the range of 100–300 *m/z*. Then, the collision energy was set to 10 to find their fragmented ions. The differences in the fragmented ions were helpful for explanation of the fragmentation pathways.

#### 3.3.3. NMR Analysis

Considering its solubility, methylene chloride-d_2_ was selected as the deuterated solvent. DNT (5 mg) was weighed into a glass vial and mixed with 1 mL of methylene chloride-d_2_ for complete dissolution. Afterward, 0.6 mL of the solution was pipetted into an NMR tube for characterization. The ^1^H spectra were collected with a 30° flip angle, 26 s relaxation delay, and zg30 pulse sequence. The pulse offset was 5.25 ppm, which was the midpoint of the selected quantitative hydrogen atoms. Data were collected with 32 repetitions of 64 K complex points. For the ^13^C spectrum, a 2 s relaxation delay and 1024 repetitions were applied. The head of the NMR probe was maintained at 296 K. TopSpin 3.1 was used for data processing.

### 3.4. Quantitative Analysis by Mass Balance

#### 3.4.1. Determination of Structurally Related Impurities

The comprehensive detection of structurally related impurities using liquid chromatographic methods is the key part of the mass balance method [30]. Their contents were closely related to industrial synthetic processes. Here, the contents of these impurities were determined by the LC-UV method. DNT stock solution was directly injected without dilution. The LC conditions were as described in our previous report [28]. All tests were carried out in triplicate and followed with solvent blank injections to avoid any carryover effect. Signal-to-noise ratios of 3 and 10 were chosen to determine the limit of detection (LOD) and the limit of quantification (LOQ) of the developed method, respectively. The contents of structurally related impurities were based on the normalized peak area of all detected peaks with the same wavelength. Because the signal intensity of impurities relied on the wavelength of UV detector, an uncertainty was introduced according to the Standard JJF 1855-2020 [31].

#### 3.4.2. Residual Organic Solvent Determination

Residual organic solvents were quantitatively determined by the PT-GC-MS method. Solid DNT was gravimetrically dissolved in 40 mL of H_2_O at a concentration of 1 mg/g. The PT sample parameters were as follows: purge temperature 20 °C, purge time 11 min, purge flow 40 mL/min, desorb preheat temperature 245 °C, desorb temperature 250 °C, desorb time 2 min, drain flow 300 mL/min, bake temperature 280 °C, and injection volume 25 mL. For the GC part, the oven temperature was programmed as follows: started from 40 °C and kept for 3 min, increased by 20 °C/min to 230 °C, and kept for 2 min. The carrier gas was helium at a flow rate of 1 mL/min through the column. For the MS part, the ion source and transfer-line temperatures were kept at 230 °C.

#### 3.4.3. Water Determination

Residual water was determined by Karl Fischer coulometric titration with a diaphragmless electrode. The method conditions were as follows: polarization current 5 μA, end voltage 100 mV, and minimum titration time 30 s. In detail, the water CRM was precisely weighed and loaded into the titration cell to verify its accuracy. Then, solid DNT (about 50 mg) was precisely weighed and added for the determination of water. Five replicates were performed for DNT. The blank value was obtained without the introduction of DNT samples and was subtracted from the measured value.

#### 3.4.4. Inorganic Impurity Determination

Both the TGA and ICP-MS methods were adopted to determine inorganic impurities. In TGA, the heating program started from 30 °C and increased to 750 °C at a rate of 30 °C/min. First, an empty pan was tested to draw a thermographic baseline of the program. Then, about 5 mg of DNT was loaded to record the weight loss during continuous combustion. The residual weight was regarded as the total of inorganic impurities. In ICP-MS, the trace levels of the 70 most common elements were individually analyzed. By this method, the DNT solution was gravimetrically prepared at about 5 mg/g and infused for analysis. The instrument parameters were the same as before [32].

### 3.5. Quantitative Analysis by qNMR

In addition to MB, qNMR provided an orthogonal approach for the purity assessment of DNT. It overcame the problem that impurities may not be detected by MB. For qNMR, an internal standard CRM (benzoic acid) was necessary for accurate quantification. In addition to the traceability to the SI unit, there should be no overlapping peaks between DNT and the internal standard. Here, DNT (7 mg) and benzoic acid (3 mg) were accurately weighed and dissolved by methylene chloride-d2. After complete dissolution, the mixture was transferred into an NMR tube. The parameters of the instrument were the same as described in Section 3.3.3.

## 4. Conclusions

The pure DNT CRM was comprehensively investigated with SI-traceable purity assessment for the first time. Qualitative characterization by IR, LC-MS/MS, and H-NMR confirmed the structural identity of DNT. Its purity was determined by both MB and qNMR, with a mass fraction of 995 mg/g and expanded uncertainty of 5 mg/g. In MB, four classes of potential impurities were extensively quantified. The mass fraction of moisture was 0.33 mg/g, while that of inorganic impurities was 0.01 mg/g, and no detectable organic solvent was detected. In qNMR, benzoic acid was used as an internal standard for direct assignment. The selected hydrogen in DNT had no overlap with any of the four structurally related impurities. The assigned purity value of the DNT can be traceable to the SI unit via an unbreakable traceability chain. This new DNT CRM will contribute to more accurate and traceable results from labs.

## Figures and Tables

**Table 1:** Uncertainty budget.

	Subitem	Value	*u*_(xi)_
MB	*P_HPLC_* (%)	99.82	0.004
	*P_W_* (mg/g)	0.33	0.190
	*P_OS_* (mg/g)	0	0
	*P_NV_* (mg/g)	0.01	0.005
	*P_MB_* (mg/g)	997.9	0.2
qNMR	*M_std_* (g/mol)	124.1368	0.00411
	*M_TMX_* (g/mol)	202.2115	0.00435
	*m_TMX_*(mg)	7.4559	0.00029
	*m_std_* (mg)	3.1701	0.00029
	*P_std_* (mg/g)	999.9	0.09
	*I_TMX_/I_std_*	1.3387	0.00036
	*P_qNMR_* (mg/g)	992.9	0.3
*P_char_*		995	3

## Data Availability

Data sharing not applicable.

## Supplementary Materials

The following supporting information can be downloaded at: https://www.mdpi.com/article/10.3390/molecules28093884/s1, Figure S1: TGA curve of DNT, Figure S2: The ^13^C spectrum of DNT, Table S1: Uncertainty from the difference in UV response factor, Table S2: Concentrations of inorganic impurities.

## Sample Availability Statement

Samples of the compounds are not available from the authors.
