# Supplementary material for: Purity Assessment of Dinotefuran Using Mass Balance and Quantitative Nuclear Magnetic Resonance
Source: Molecules. 2023 May 4;28(9):3884. doi: 10.3390/molecules28093884 (PMC10179808; doi:10.3390/molecules28093884)
Supplement: Supplementary file 1 [file molecules-28-03884-s001.zip › molecules-2317595-supplementary.pdf]

Supplementary Information

Purity assessment of dinotefuran using mass balance and quantitative nuclear magnetic resonance

Xianjiang Li[1]*, Wei Zhang[1], Xiao Li[1], Shukun Zhou[1], Mengling Tu[1], Yunxiao Zhu[1,2], Hongmei Li[1]

[1]Key Laboratory of Chemical Metrology and Applications on Nutrition and Health for State Market Regulation, Division of Metrology in Chemistry, National Institute of Metrology, Beijing 100029, China

[2]State Key Laboratory of Heavy Oil Processing, College of Chemical Engineering and Environment, China University of Petroleum, Beijing 102249, PR China

*Corresponding author: Xianjiang Li, Email: lixianjiang@nim.ac.cn;

Tel: +86-10-64524727, Fax: +86-10-64524783

Address: Division of Metrology in Chemistry, National Institute of Metrology, No. 18 East Road of North 3rd Ring, Chaoyang District, Beijing 100029, China

ORCID: Xianjiang Li (0000-0003-0310-0376)

Wei Zhang (0000-0001-8170-3178)

Xiao Li (0000-0003-1588-1675)

Shukun Zhou (0000-0002-5348-0218)

Mengling Tu (0000-0002-2077-8528)

Yunxiao Zhu (0000-0003-1585-7333)

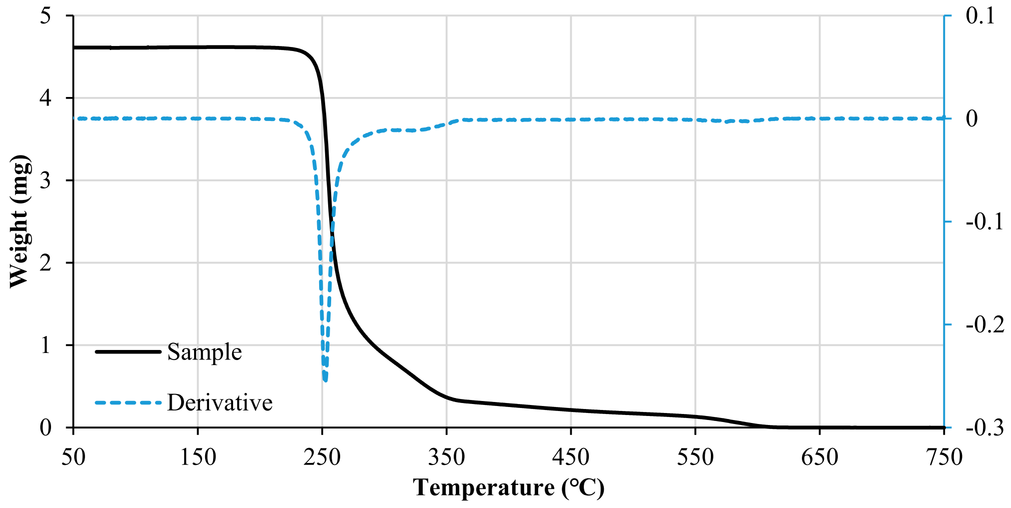

Figure S1. TGA curve of DNT

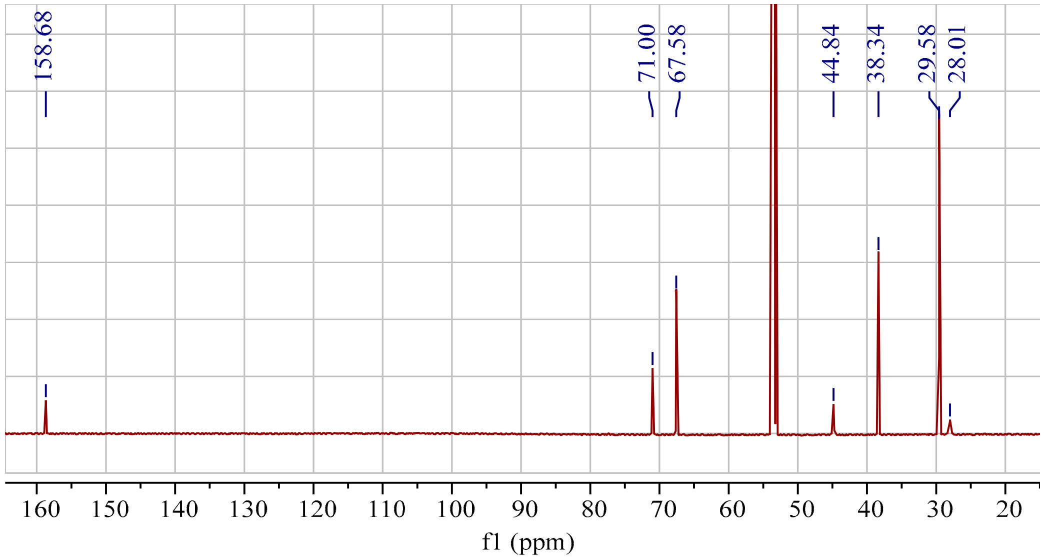

Figure S2. ^{13}C spectrum of DNT

Table S1. Uncertainty from the difference in UV response factor.

Wavelength (nm)	DNT (μV·s)	Impurity 1 (μV·s)	Impurity 2 (μV·s)	Impurity 3(μV·s)
254	7040094	2495	6254	3598
268	9881402	3482	9339	5140
269	9856533	3250	9406	5219
275	9072675	2756	8325	4983
254	7040094	2495	6254	3598
u_{2-i}	/	0.0023%	0.0000%	0.0000%
u_2	0.0014%			

Table S2 Concentrations of inorganic impurity

Elements	Molecular weight (g/mol)	Concentration (ng/L)	Elements	Molecular weight (g/mol)	Concentration (ng/L)
Li	7	/	In	115	/
Be	9	/	Sn	118	/
B	11	/	Sb	121	/
Na	23	/	Te	125	/
Mg	24	/	I	127	/
Al	27	/	Cs	133	0.01
Si	28	/	Ba	137	37.48
K	39	/	La	139	/
Ca	44	/	Ce	140	/
Sc	45	/	Pr	141	/
Ti	47	/	Nd	146	/
V	51	0.02	Sm	147	/
Cr	53	/	Eu	153	/
Mn	55	0.05	Gd	157	/
Fe	57	/	Tb	159	/
Co	59	/	Dy	163	/
Ni	60	/	Ho	165	/
Cu	63	0.04	Er	166	/
Zn	66	/	Tm	169	/
Ga	69	1.99	Yb	172	/
Ge	72	/	Lu	175	/
As	75	/	Hf	178	/
Se	82	/	Ta	181	/
Br	79	/	W	182	/
Rb	85	/	Re	185	/
Sr	88	/	Os	189	/
Y	89	/	Ir	193	/
Zr	90	/	Pt	195	/
Nb	93	0.01	Au	197	/
Mo	95	/	Hg	202	/
Ru	101	/	Tl	205	/
Rh	103	0.19	Pb	208	/
Pd	105	/	Bi	209	/
Ag	107	/	Th	232	/
Cd	111	/	U	238	/
Total concentration (ng/L)			39.8		
DNT concentration (ng/L)			4228000		
Inorganic impurity (mg/g)			0.01		
